# Circulating Tumour Cells as an Independent Prognostic Factor in Patients with Advanced Oesophageal Squamous Cell Carcinoma Undergoing Chemoradiotherapy

**DOI:** 10.1038/srep31423

**Published:** 2016-08-17

**Authors:** Po-Jung Su, Min-Hsien Wu, Hung-Ming Wang, Chia-Lin Lee, Wen-Kuan Huang, Chiao-En Wu, Hsien-Kun Chang, Yin-Kai Chao, Chen-Kan Tseng, Tzu-Keng Chiu, Nina Ming-Jung Lin, Siou-Ru Ye, Jane Ying-Chieh Lee, Chia-Hsun Hsieh

**Affiliations:** 1 Circulating Tumour Cell Lab, Division of Medical Oncology, Department of Internal Medicine, Chang Gung Memorial Hospital at Linkou, College of Medicine, Chang Gung University, Taoyuan, Taiwan; 2 Graduate Institute of Biochemical and Biomedical Engineering, Chang Gung University, Taoyuan, Taiwan; 3 Department of Chemical Engineering, Ming Chi University of Technology, New Taipei City, Taiwan; 4 Division of Endocrinology and Metabolism, Department of Internal Medicine, Taichung Veterans General Hospital, Taichung, Taiwan; 5 Department of Public Health, College of Public Health, China Medical University, Taichung, Taiwan; 6 Department of Medical Research, Taichung Veterans General Hospital, Taiwan; 7 Department of Oncology–Pathology, Karolinska Institutet, Stockholm, Sweden; 8 Cancer Center Karolinska, Karolinska University Hospital, Stockholm SE-17176, Sweden; 9 Northern Institute for Cancer Research, School of Medicine, Newcastle University, Newcastle, United Kingdom; 10 Division of Thoracic Surgery, Chang Gung Memorial Hospital, College of Medicine, Chang Gung University, Taoyuan, Taiwan; 11 Department of Radiation Oncology, Chang Gung Memorial Hospital, College of Medicine, Chang Gung University, Taoyuan, Taiwan; 12 Department of Chemical and Materials Engineering, Chang Gung University, Taoyuan, Taiwan

## Abstract

The role of circulating tumour cells (CTCs) in advanced oesophageal cancer (EC) patients undergoing concurrent chemoradiotherapy (CCRT) remains uncertain. A negative selection protocol plus flow cytometry was validated to efficiently identify CTCs. The CTC number was calculated and analysed for survival impact. The protocol’s efficacy in CTC identification was validated with a recovery rate of 44.6 ± 9.1% and a coefficient of variation of 20.4%. Fifty-seven patients and 20 healthy donors were enrolled. Initial staging, first response to CRT, and surgery after CRT were prognostic for overall survival, with P values of <0.0001, <0.0001, and <0.0001, respectively. The CTC number of EC patients is significantly higher (P = 0.04) than that of healthy donors. Multivariate analysis for disease-specific progression-free survival showed that surgery after response to CCRT, initial stage, and CTC number (≥21.0 cells/mL) played independent prognostic roles. For overall survival, surgery after CCRT, performance status, initial stage, and CTC number were significant independent prognostic factors. In conclusion, a negative selection plus flow cytometry protocol efficiently detected CTCs. The CTC number before CCRT was an independent prognostic factor in patients with unresectable oesophageal squamous cell carcinoma. Further large-scale prospective studies for validation are warranted.

Oesophageal cancer (EC) is the 7^th^–8^th^ most common cancer and is the 7^th^ most common cause of death related to cancer in the United States^1^ and Europe^2^,^3^, and also in Asia, including Taiwan^4^. There are two main histological types of EC: squamous cell carcinoma (ESCC) and adenocarcinoma (EAC). Currently, the former type accounts for the majority of cases of EC in African American, southern European, and Asian populations, whereas the incidence of the latter has tended to show gradual increases in the United States and northern Europe^1^,^2^,^3^,^4^. In EC that is unresectable or at locally advanced stages, regardless of the type, concurrent chemoradiotherapy (CCRT) has been the golden standard in treatment for decades^5^,^6^. Even in metastatic settings, palliative CCRT remains the main method of relieving symptoms resulting from cancer^7^,^8^. In recent years, several prognostic factors, such as the decreased number of lymph nodes after CCRT^9^, pathologic complete remission after CCRT and surgery^10^, and a history of heavy smoking^11^, have been found to be clinically prognostic in EC patients scheduled for CCRT. However, several biomarkers, such as microRNA (miRNA)^12^,^13^, NY-ESO-1 autoantibody^14^, and anti-P16 antibody^15^, are under investigation and awaiting large-scale clinical trials for evaluation.

Circulating epithelial or tumour cells (CECs or CTCs), identified and of interest since 1869^16^, are defined as cells expressing epithelial cell surface markers and/or tumour specific marker(s) and must simultaneously be excluded from red/white blood cells (RBCs/WBCs) in the circulation. These cells have been thought to be live cells shed from the primary tumour mass, which are cultivable^17^,^18^, have the potential to metastasise into distant organs^19^, promote thrombosis^12^, acquire resistance to anticancer drugs^20^,^21^,^22^,^23^, and have been proven to be prognostic and predictive in patients with various kinds of solid tumours^24^,^25^,^26^,^27^,^28^,^29^. Even more, CTCs could also potentially guide anticancer therapies^22^,^30^. Development of a reliable method of detection or isolation of CTCs could represent a good biomarker or predictor before the initiation of anticancer treatment in cancer patients. The major limitation in the efficacy of CTC isolation has spurred advances in nanoscience^31^, biochips^18^,^32^, physiology^31^,^33^, chemistry, and novel surface markers^34^,^35^,^36^, as well as many new methods or devices^37^. Although the CellSearch^®^ system was developed and approved by the US Food and Drug Administration (FDA) in 2004, there is still no standard method or protocol to identify or isolate CTCs because of the relatively low efficiency of detection to date. In our opinion, a cheap and easy-to-access protocol or device is urgently needed.

For EC patients, the role of CTCs remains unclear in the literature. Therefore, to elucidate the clinical relevance of CTCs in patients with locally advanced ESCC, which is the most common type of EC at diagnosis, we prospectively designed and conducted a trial in a single medical centre in Taiwan. In addition, we attempted to report the efficacy of a relatively easy-to-perform method for CTC detection to enhance the advances in the field of CTCs.

## Material and Methods

### Study Design

The study was designed to be a prospective observational study. We aimed to elucidate the clinical significance of baseline CTCs before CCRT of unresectable ESCC patients. To determine a cutoff of CTC number for further survival analysis, we designed to use the cutoff found by ROC curves with Youden test (Supplementary Table S1) in EC patients (n = 57) and healthy donors (n = 20) in this pilot study. The endpoints of the study were to find the correlations among baseline CTCs, progression-free survival (PFS), and overall survival (OS). Following treatment response, surgery, disease progression and death from any causes were documented for survival analysis. The analysis was done only after when more than half of the events have occurred. A score combining treatment response and baseline CTC categories was designed to be analysed for its prediction ability of cancer death.

### Patient Enrolment

This study was conducted in a single medical centre, Chang Gung Memorial Hospital, in Linkou, Taiwan. The study protocol was approved by the Institutional Review Board of Chang Gung Memorial Hospital (approval ID: 101-2161C). All patients provided written informed consent for the ethically approved protocols. Eligible patients with histologically or cytopathologically confirmed ESCC were all medically unfit for surgery or had surgically unresectable, locally advanced (stage IIb–IV, American Joint Committee on Cancer [AJCC] 7^th^ edition), or metastatic cancer at initial presentation. Other enrolment criteria included: (1) age ≥ 20 years old; (2) patients who could understand and sign the informed consent by their own will; and (3) patients with adequate liver and renal function and WBC counts for anticancer therapies, especially for CCRT. Patients with synchronous cancer or prior cancers within 5 years, except for non-melanoma skin cancers and *in situ* cervical cancers, were excluded at enrolment. Disease staging and management followed the standard treatment protocols according to institutional guidelines. Blood samples were drawn within 7 days before the first dose of chemotherapy. Study results were reported following the REporting recommendations for tumour MARKer prognostic studies (REMARK) guidelines^38^. All the methods were carried out in accordance with the relevant guidelines, including any relevant details. Examinations for initial staging and response evaluation included computed tomography (CT), pan-endoscopy, endoscopic ultrasonography (EUS), positron emission tomography (PET), and/or bronchoscopy, except in patients who initially had metastatic lesions on CT scans. EUS was performed with an ultrasonic miniprobe (UM2R/12 MHz or UM3R/20 MHz; Olympus, Inc., Tokyo, Japan). Following the standard treatment guidelines, CCRT and/or surgery were scheduled and delivered by the medical oncologists, radiation oncologists, and chest surgeons on the oesophageal tumour board at Chang Gung Memorial Hospital. Based on the Response Evaluation Criteria in Solid Tumours (RECIST) version 1.0 guidelines, the treatment response, including complete remission (CR), partial response (PR), stable disease (SD), and progressive disease (PD), was determined by the multidisciplinary oesophageal tumour board.

### Chemotherapy Regimens of Chemoradiotherapy

Two chemotherapy regimens of CCRT were scheduled. One was 5-fluorouracil (1,000 mg/m^2^/day, administered as a continuous infusion over a period of 96 h on days 1–4 and 29–33) plus cisplatin (75 mg/m^2^, administered as an intravenous infusion for 3 h on days 1–29), which was called the PF regimen. The other regimen was carboplatin (area under the curve of 2, weekly) plus paclitaxel (50–60 mg/m^2^, weekly) for 6 consecutive doses, which was called the TC regimen. Radiotherapy was administered either sequentially to PF chemotherapy on days 8–29 to a total dose of 30 Gy (200 cGy/fraction) or concurrently with TC chemotherapy to a total dose of 41.4 Gy (180 cGy/fraction).

### Clinical Assessment of Chemoradiotherapy Effectiveness and Consolidation Therapy for Patients Who Did Not Undergo Oesophagectomy

The clinical response to CCRT was determined based on the results of endoscopy and imaging findings (CT and oesophagography) at 5–6 weeks post-treatment. As of 2007, PET scans were used for both staging and restaging workups. The response was considered complete (cCR) in the presence of the following criteria: (1) no evidence of disease on CT scans and no increased tracer uptake on PET images; and (2) no stricture, no residual tumour nor ulcer identified by panendoscopy, and negative biopsy results. Endoscopic findings in cCR patients were further classified into three categories: (1) Scar: healing ulcer; (2) Other finding: other abnormal mucosal findings (e.g., mucosa tag, polypoid lesion, granular protruded lesions, erosion, and lugol-voiding lesions); and (3) Normal: patients who did not show any mucosal abnormality. In the absence of contraindications, oesophagectomy was scheduled in all patients. Eligibility for surgery was based on the following: (1) medical fitness for surgery, with absence of liver cirrhosis and/or heart failure (New York Heart Association class III or IV); (2) absence of tracheoesophageal fistula; and (3) no evidence of recurrent laryngeal nerve invasion. For patients who refused scheduled surgery after neoadjuvant chemoradiotherapy (nCRT), another course of CRT, consisting of PF or TC chemotherapy, was given as consolidation therapy. The total radiation dose was 60 Gy.

### Experiments for Efficiency of Detection and Patients’ Sample Analysis

**Figure 1:**
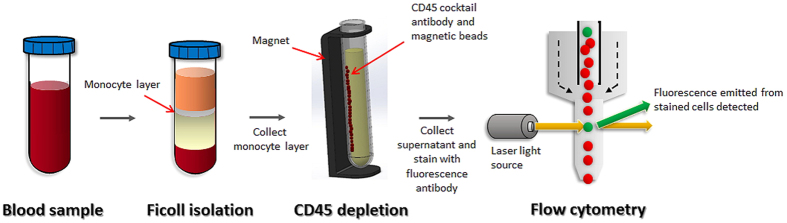
Illustration of circulating tumour cell (CTC) detection protocol.

CTC analysis was performed using a protocol with combined negative selection and positive detection strategies. Briefly, the methods were (1) a negative selection protocol for effective RBC and leukocyte depletion with Ficoll isolation and a CD45 depletion kit; and (2) flow cytometry to quantitatively identify the number of CTCs. The concept of the protocol is illustrated in Fig. 1a. For control and validation experiments with head and neck cancer cell lines, the details were described in the Supplementary file.

### Statistical Analysis

The numbers of pre-CCRT CTCs in advanced EC patients and healthy donors were compared using box plots and the Mann-Whitney U test with two-sided significance. Factors influencing the survival of patients with EC were assessed by univariate and multivariate Cox proportional hazards regression analysis. Parameters with significance in univariate analysis were subjected to multivariate Cox regression analysis. Disease-specific PFS was calculated from the date of CTC sampling, within 7 days before the start of CCRT, to the date of cancer-specific progression. OS was defined as the period from the date of CTC sampling to the date of death from any cause. The circulating tumour cell plus response (CTCR) score is defined as the summation of the CTC score (zero for CTC number less than 21.0 cells/mL; 1 for CTC number ≥ 21.0 cells/mL) and the score of the CCRT response (zero for CR; 1 for PR; 2 for SD; and 3 for PD). Statistical analysis was performed using SPSS for Windows (version 18, SPSS Inc., Chicago, IL, USA). A P value of 0.05 was considered statistically significant.

## Results

### The Protocol Efficiency, Recovery Rate, and Purity

The efficacy of the protocol in identifying CTCs was validated with a recovery rate of 44.6 ± 9.1% and a % coefficient of variation (CV) of 20.4% in cell line spiking experiments (n = 20). Blood samples from healthy individuals were used as a control group, and the recovery rate of flow cytometry was 71.1 ± 12.0% with a %CV of 16.8% (experiment n = 43, 20 individuals in total).

### Patient Enrolment

Between December 2012 and December 2014, a total of 57 patients were enrolled after detailed introduction of the trial design, scientific goals, and inconvenience/risks of participation in the Division of Haematology-Oncology, Chang Gung Memorial Hospital in Linkou. Meanwhile, 20 healthy donors were also recruited as controls for the CTC analysis. Basic characteristics of the enrolled patients are shown in Table 1. Clinical information and survival data were updated until August 2015. The median age of the patients was 54 (36–78) years old. The majority of tumours were of the moderately differentiated type (68.4%), and the most frequent tumour locations in our population were the middle (36.8%) and upper part (36.1%) of the oesophagus. In addition, most patients scored an Eastern Cooperative Oncology Group (ECOG) performance status of 0–1 (61.4%), whereas the other 38.6% scored ≥2.

### The Impacts of Initial Staging and Surgery After Chemoradiotherapy

**Figure 2:**
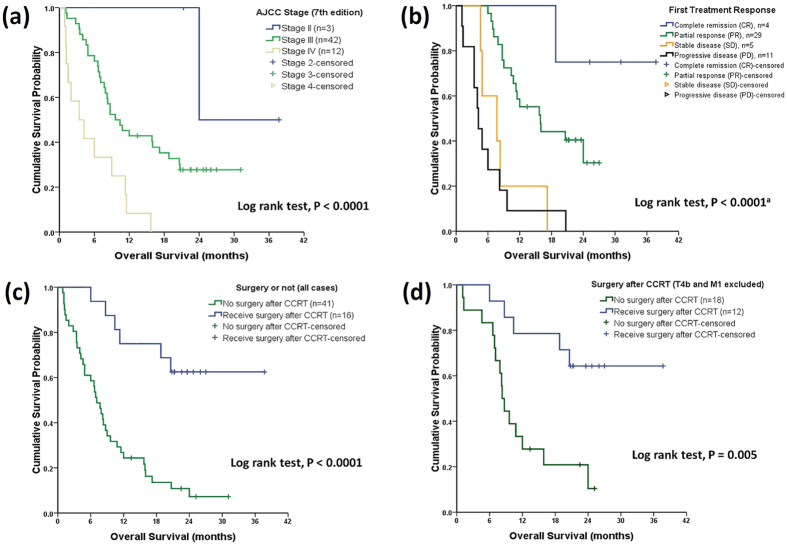
The correlations among tumour staging, response, surgery, unresectable status and survival. Panel A demonstrates that initial TNM staging correlates with overall survival (OS). Treatment response after concurrent chemoradiotherapy (CCRT) also correlates with OS (Panel B). Residual tumour with or without surgery is also highly prognostic for OS (Panel C); patients with initial T4b and M1 status are excluded (Panel D).

Figure 2a–d demonstrates the prognostic survival impact of the initial AJCC (7^th^ edition) staging, first treatment response to CRT, surgery after CRT, and undergoing surgery, with the exception of patients with initial T4b and M1 status, with P values of <0.0001, <0.0001, <0.0001, and 0.005, respectively. In patients with imaging CR, PR, SD, and PD to CCRT, the median OS values were not reached, 15.9, 7.7, and 4.2 months, respectively, with a log rank test P value of <0.001.

### Clinical Relevance of Circulating Tumour Cells Before Chemoradiotherapy

**Figure 3:**
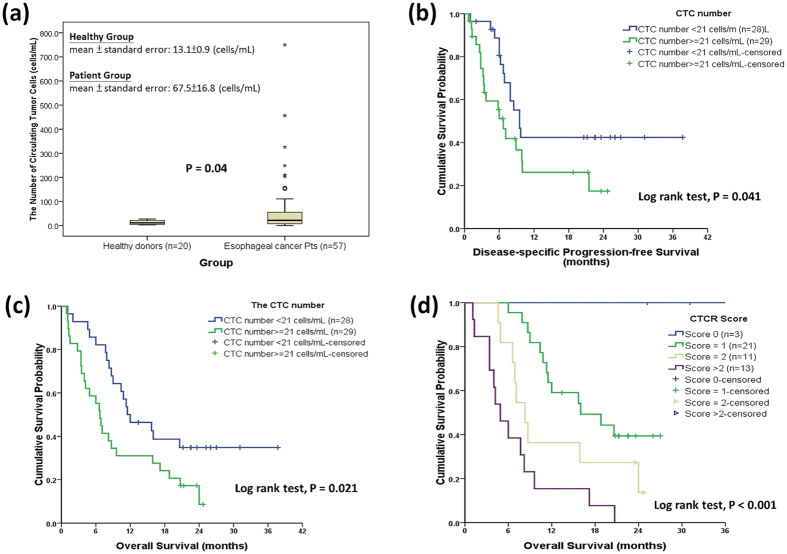
The number of circulating tumour cells (CTCs) can differentiate oesophageal cancer patients and its impact on survival. The method of circulating tumour cell (CTC) detection can differentiate healthy individuals from patients with advanced oesophageal cancer with a P value of 0.04 using the Mann-Whitney U test (Panel A). Panels B and C show that patients with a lower pre-treatment CTC number have longer disease-specific progression-free survival or overall survival. Given the CTC status (score zero for CTC number less than 21.0 cells/mL; 1 for CTC number ≥ 21.0 cells/mL) and response after concurrent chemoradiotherapy (CCRT, score zero for complete remission; 1 for partial response; 2 for stable disease, and 3 for progressive disease), the summation of the CTC score and the score of response to CCRT is defined as the circulating tumour cell plus response (CTCR) score, and this score highly correlates with overall survival with a log rank test P value of <0.0001.

The CTC numbers of patients with EC (n = 57) were significantly higher (Mann-Whitney T test; P = 0.04) than those of healthy donors (n = 20) (Fig. 3a). A cutoff of 21.0 cells/mL with a sensitivity of 52.6% and a specificity of 80.0% was obtained by ROC curve (details in Supplementary Table S1) to differentiate EC patients from healthy individuals. After cox-proportional hazards model was examined, the following analysis showed that the CTC number before CCRT can serve as a prognostic factor for disease-specific PFS and OS in patients with advanced squamous cell carcinoma with log rank test P values of 0.041 and 0.021, respectively (Fig. 3b,c). In multivariate analysis for disease-specific progression, surgery after response to CCRT, initial AJCC TNM stage, and CTC number (≥21.0 cells/mL) were found to be independent prognostic factors with P values of 0.001, <0.001, and 0.004, respectively (Table 2). In multivariate analysis for OS, surgery after response to CCRT, ECOG performance status, initial TNM stage (AJCC 7^th^ edition), and CTC number were found to be independent prognostic factors with P values of <0.001, <0.001, 0.003, and 0.028, respectively (Table 2). Cancer location and chemotherapy regimen did not impact the OS (P = 0.515 and 0.136, respectively; Supplementary Figure S1A–C). The baseline CTC number group is predictive for response to CCRT and locoregional or distant failure with P values of 0.001 and 0.027, respectively, by the chi-square test (Supplementary Table S2). In further analysis, the CTCR score was found to be able to significantly differentiate patients with advanced EC into groups with distinct OS after first response evaluation (Fig. 3d and Supplementary Figure S1D). However, there was no clear correlation among CTC number and clinical T stage (P = 0.088), N stage (P = 0.164), and initial M status (P = 0.400) by the chi-square test (Table not shown).

## Discussion

This study firstly demonstrated an overall recovery rate of 44.6 ± 9.1% and a %CV of 20.4% using a simple, cheap, and widely available CTC analysis method with Ficoll isolation and CD45 depletion by magnetic beads followed by flow cytometry for detection. The detection rate of CTCs was 100.0% and a cutoff of 21.0 cells/mL was obtained by ROC curve (P = 0.04). Not surprisingly, the initial staging, surgery after CCRT, and the response to CCRT contributed to the OS of ESCC patients with statistical significance. (P < 0.0001, <0.0001, and <0.0001, respectively) Taking into consideration of all the above factors, the pre-CCRT CTC number showed its independent prognostic impact on disease-specific PFS and OS with hazard ratios (HRs) (95% confidence interval [CI]) of 3.113 (1.427–6.791) and 1.002 (1.000–1.004), respectively. In addition, the CTCR score, which was first proposed in the literature, could clearly separate the OS with statistical significance (P < 0.001), by considering both the CTC category (low, high) and responses (CR, PR, SD + PD). The CTCR score might be able to provide group selection in future clinical trials. To the best of our knowledge, this is the first report using a negative selection strategy combined with flow cytometry to perform CTC analysis in advanced ESCC patients who underwent CCRT that proved the independent prognostic role of CTCs.

In the literature, there are only a few studies that address the role of CTCs in EC patients. Table 3 summarizes the most important information from these studies. In 2002, Koike, M. *et al.* reported the first work using reverse transcription-polymerase chain reaction (RT-PCR) to detect tumour-specific carcinoembryonic antigen (CEA) messenger RNA (mRNA) in the circulation and concluded that the CEA mRNA measured by RT-PCR could be more sensitive than conventional serum tumour markers (CEA and squamous cell carcinoma [SCC])^39^. Also utilizing RT-PCR, Nakashima, S. *et al.* found in 2003 that CEA mRNA detection could predict cancer recurrence^40^. In the following year, Ito *et al.* found that CEA combined with cytokeratin 20 (CK20) mRNA (CEA assay) could also predict tumour recurrence better than serum tumour markers^41^. In 2007, Ikoma, D. *et al.* used multiple mRNAs, including CEA, p16, E-cadherin, and retinoic acid receptor (RAR) to monitor EC cancer after curative surgery. In the same year, Liu, Z. *et al.* used CEA mRNA from peripheral blood cells in a Chinese population to prove that the CEA mRNA expression detected by RT-PCR could predict distant metastasis in EC patients who underwent curative surgery^42^. In addition to patients with resectable tumours, Yin, X. D. *et al.* chose EC patients scheduled to undergo radical radiotherapy, and reported that the detection of CEA+CK19+ survivin mRNA by RT-PCR could be a promising biomarker for radiation efficiency and assessment of prognosis. Until 2012, RT-PCR for tumour-specific mRNA was the main method for CTC detection. However, cancer cells were not actually captured in these studies, and cancer-specific mRNA expression might not truly correlate with the cancer cell count in the circulation. In 2014, Bobek, V. *et al.* from the Czech Republic reported on an interesting device utilising a size-based mechanism to literally capture CTCs^43^. The report concluded that the method could capture CTCs and prove that these cells are alive for further cultivation. This was a novel and important finding, but no clinical relevance was reported with the use of this device. The CellSearch^®^ system (Janssen Diagnostics, LLC, Raritan, NJ, USA), an FDA-approved device using a positive selection strategy to identify CTCs, was used by three groups, Matsushitam *et al.* Reeh *et al.* and Tanaka *et al.*, and the correlations with treatment response and an independent prognostic role of CTCs were impressive^26^,^44^,^45^. However, one of the drawbacks of the Cellsearch^®^ system was its known detection rate, suggesting a possible loss of CTC information. Furthermore, performing CTCs analysis with the CellSearch^®^ system is relatively costly and device-dependent. Theoratically, our method, utilising negative depletion of CD45^+^ cells and positive selection of EpCAM and cytokeratins with flow cytometry, is capable of providing clinicians and medical researchers a much easier and cheaper platform to conduct CTC-related clinical trials. Moreover, our study focused on patients with unresectable or metastatic status, a group seldom investigated in the literature.

One subject we should particularly discuss is that the survival of enrolled patients in our study was relatively short when compared with that of the general EC population. Several plausible reasons include, firstly, the fact that the study only enrolled Asian patients with ESCC and that 38.6% of the patients were classified as ECOG PS ≥ 2, which means that the status of the population could be worse than that of general, locally advanced EC patients. Secondly, there are some differences amongst Western and Asian patients with EC, including major histological types, risk factors, male-to-female ratio, and clinical outcomes. In comparison with Western countries, the major histology in Asian EC patients is still SCC, and this accounts for 91.5% of EC patients in Taiwan^46^, while in some areas of the United States, up to 60% of patients are diagnosed with AC^1^. Poor outcomes in Taiwanese patients could be explained as a result of relatively low socioeconomic status and exposure to multiple carcinogens, including cigarettes, alcohol, and betel nut chewing^47^. Thirdly, the male-to-female ratio (27.5 to 1) in our study is not comparable to that of the general EC population, which might partially explain the short survival in this study. More specifically, the male-to-female ratio is approximately 13.57 to 1^4^ according to the Taiwan Cancer Registry database, whereas a ratio of 3 to 1 is commonly noted in Western countries^1^,^2^. The phenomenon of male populations harbouring worse outcomes than those for women has also been noticed in some southern European countries^2^. These reasons could explain the short survival, but would not confuse the role of CTCs after statistical adjustment.

Although the current study successfully demonstrated the correlations among the number of CTCs, the disease-specific PFS and OS, and the cutoff of CTCs with clinical impact, these could change with different methods of CTC detection. In fact, different methods would yield different recovery rates and purity. We should note that the CTC number obtained by different methods should not be compared for clinical significance. Furthermore, the cutoff value with one type of CTC detection method is different from another. That is, the concept of a “low” or “high” CTC number would be relatively adequate rather than setting a definite CTC number cutoff for all solid tumours. In the present study, an easy-to-perform, controlled, cheap, and commonly available method is proposed to help foster the availability of CTC studies in every medical laboratory. Sample loss could be a problem with this method, but this could be overcome through careful procedures conducted by well-trained technicians. Although we found a cutoff to separate EC patients into two groups with survival difference, the relatively small sampling size limited the accuracy of the cutoff in this pilot study. In the near future, a satisfactory cutoff value will possibly be decided upon after the collection of large-scale information using one single method with similar controls. Possible reasons that could explain the false positivity of this method include the background noise or non-significant epithelial cells in the circulation, and further long-term observations for these patients are still needed. Another confirmation method may be possible with the sequencing of cancer-specific DNA mutations on CTCs to avoid bias. Although the technique of DNA mutation detection on CTCs has been proposed, it still seems to be highly technique-dependent^48^,^49^.

There are still some limitations of this study to discuss. Firstly, patients with T4b cancer and those with cervical EC were enrolled in this study, which could contribute some bias. These patients would not undergo surgery due to their disease status even with the achievement of PR after CCRT. One other problem was the fact that some patients, mostly of cT4b status, who died of sudden massive tumour bleeding and/or sepsis from tracheoesophageal fistula during CCRT, caused shortening of the survival in this study. Secondly, the Cellsearch^®^ system can generate a detection rate (≧5 cells/7.5 mL blood) of 0~48%^50^, a recovery rate of 80~82%^51^ and an inter-laboratory % CV of 45~64%^52^. We admit that our recovery rateof our protocol was lower than Cellsearch^®^; however, our protocol was designed to be very easily obtained and performed. The relatively low recovery was probably resulted from Ficoll separation procedure which lost a part of CTCs^53^, but it could separate red blood cells completely and allow further molecular analysis to proceed. Nevertheless, given consideration of these conditions, CTCs could still play an important role in patients with EC. Further studies on females or patients with EAC in the Asian population are warranted.

In conclusion, we attempted to develop, validate, and report on an easy-to-use platform for CTC analysis that is mostly available in medical laboratories. The CTC number before CCRT was proven to be an independent prognostic factor in patients with unresectable ESCC. However, as with many proof-of-concept reports, further large-scale prospective studies to determine a better cutoff value are warranted.

## Additional Information

**How to cite this article**: Su, P.-J. *et al.* Circulating Tumour Cells as an Independent Prognostic Factor in Patients with Advanced Oesophageal Squamous Cell Carcinoma Undergoing Chemoradiotherapy. *Sci. Rep.* **6**, 31423; doi: 10.1038/srep31423 (2016).

## Supplementary Material

Supplementary Information

## Figures and Tables

**Table 1:** Basic Characteristics of Enrolled Patients.

	n	%
**Age,** median (range), years	54 (36–78)	
**Gender,** M/F	55/2	
Histology		
Squamous cell carcinoma	57	100.00%
ECOG PS		
0–1	35	61.4%
≥2	22	38.6%
Differentiation of Cancer		
Well differentiated	3	5.3%
Moderately differentiated	39	68.4%
Poorly differentiated	15	26.3%
Tumour Location		
Cervical	5	8.8%
Upper third	20	35.1%
Middle third	21	36.8%
Lower third	11	19.3%
Chemotherapy Regimen of CCRT		
Cisplatin plus 5-fluorouracil	37	64.9%
Carboplatin plus paclitaxel	20	35.1%

Abbreviations: M/F, male/female; ECOG PS, Eastern Cooperative Oncology Group performance status; CCRT, concurrent chemoradiotherapy.

**Table 2:** Univariate and Multivariate Analysis for Survivals.

Prognostic Factors	Disease-specific Progression-free Survival						Overall Survival					
	Univariate			Multivariate			Univariate			Multivariate		
	HR for PD	(95% CI)	P value	HR for PD	(95% CI)	P value	HR for death	(95% CI)	P value	HR for death	(95% CI)	P value
Age, (yr)												
≥50 vs. <50	1.108	(0.558–2.199)	0.770				0.930	(0.510–1.697)	0.814			
Surgery after CCRT												
Yes vs. No	0.142	(0.049–0.413)	<0.001	0.160	(0.052–0.486)	0.001	0.189	(0.079–0.455)	<0.001	0.19	(0.076–0.472)	<0.001
ECOG PS												
0–1 vs.≥ 2	0.604	(0.286–1.272)	0.185				0.296	(0.157–0.556)	<0.001	0.276	(0.138–0.553)	<0.001
TNM Stage (7^th^ ed.)			<0.001			<0.001			0.001			0.003
Stage II	0.072	(0.009–0.591)	0.014	0.041	(0.004–0.388)	0.005	0.077	(0.010–0.612)	0.015	0.112	(0.013–0.944)	0.044
Stage III	0.210	(0.094–0.470)	<0.001	0.186	(0.077–0.452)	0.000	0.310	(0.154–0.626)	0.001	0.306	(0.147–0.636)	0.002
Stage IV	reference			reference			reference			reference		
CTC number (cells/mL)												
≥21.0 vs. <21.0	2.022	(1.010–4.049)	0.047	3.113	(1.427–6.791)	0.004	2.661	(1.375–5.150)	0.024			
CTC number (cells/mL)	1.002	(0.999–1.004)	0.139				1.003	(1.001–1.005)	0.002	1.002	(1.000–1.004)	0.028

Abbreviations: HR, hazard ratio; CI, confidence interval; PD, progressive disease; CCRT, concurrent chemoradiotherapy; ECOG PS, Eastern Cooperative Group performance status; CTC, circulating tumour cells.

**Table 3:** Mini-review of Circulating Tumour Cells in Oesophageal Cancer.

Author	Year	Country	Patient Group	N (Detection Rate)	Methods of CTC Analysis	Major Results
Molecular detection						
Koike, M. *et al.*	2002	Japan	Resectable, mainly	43 (53.5%)	RT-PCR for Np63 mRNA	More sensitive than SCC and CEA
Nakashima, S. *et al.*	2003	Japan	Resectable	54 (54.7%)	RT-PCR for CEA mRNA	More predictive of tumour recurrence than serum tumour markers
Ito, H. *et al.*	2004	Japan	All stages	28 (25.0–57.9%)	RT-PCR for CEA+CK20 mRNA (CEA assay)	A reliable means of predicting early recurrence.
Ikoma, D. *et al.*	2007	Japan	Resectable	44 (53.0%)	RT-PCR for CEA, p16, E-cadherin, RAR mRNA	Can serve as complementary markers for screening and monitoring oesophageal cancer patients
Liu, Z. *et al.*	2007	China	Resectable	53 (28.3–60.4%)	RT-PCR for CEA mRNA	Operation results in tumour cell dissemination and significant increase of CTCs in peripheral blood, which is related to the developed metastasis
Yin, X. D. *et al.*	2012	China	Radical radiotherapy	72 (38.9–54.2%)	RT-PCR for CEA+CK19+survivin mRNA	Positive detection of CTCs in patients with ESCC after radiotherapy may be a promising biomarker for radiation efficiency and assessment of prognosis
Physical Strategy (Size or Filter)						
Bobek, V. *et al.*	2014	Czech	Resectable	43 (62.8%)	size-based filtration, CK18(+)	Successful culturing of oesophageal cancer CTCs
Immunomagnetic Isolation (Positive or Negative Strategy)						
Matsushitam D. *et al.*	2015	Japan	Resecatble and unresecatble cases, CT or CCRT	90 (27.8%)	CellSearch (positive selection)	CTCs correlate to treatment response, prognostic factor(+)
Reeh, M. *et al.*	2015	Germany	Resectable, no CT or CCRT	123 (18%)	CellSearch (positive selection)	Independent, prognostic indicators of patients’ outcome in EC; implementation of CTCs may improve accuracy of preoperative staging in EC
Tanaka *et al.*	2015	Japan	Unresectable, CT or CCRT	38 (50%)	CellSearch (positive selection)	CTCs can be useful for predicting the survival and for monitoring the treatment response
Su, P. J. *et al.*	2016	Taiwan	Unresectable, All CCRT	57 (100.0%)	Negative selection (CD 45 depletion) + Flow cytometry	Surgery after response to CCRT, ECOG PS, initial TNM stage, and CTC number are independent prognostic factors

Abbreviations: RT-PCR, reverse transcription polymerase chain reaction; CEA, carcinoembryonic antigen; mRNA, messenger ribonucleic acid; CK, cytokeratin; CTC, circulating tumour cells; ESCC, oesophageal squamous cell carcinoma; CT, chemotherapy; CCRT, concurrent chemoradiotherapy; EC, oesophageal carcinoma; ECOG PS, Eastern Cooperative Oncology Group performance status.
