# Supplementary material for: Circulating Tumour Cells as an Independent Prognostic Factor in Patients with Advanced Oesophageal Squamous Cell Carcinoma Undergoing Chemoradiotherapy
Source: Sci Rep. 2016 Aug 17;6:31423. doi: 10.1038/srep31423 (PMC4987675; doi:10.1038/srep31423)
Supplement: Supplementary Information [file srep31423-s1.pdf]

Supplementary Information

TITLE OF THE MANUSCRIPT:

Circulating Tumour Cells as an Independent Prognostic Factor in Patients with Advanced Oesophageal Squamous Cell Carcinoma Undergoing Chemoradiotherapy

AUTHORS:

Po-Jung Su[1‡], Min-Hsien Wu[1,2‡], Hung-Ming Wang[1‡], Chia-Lin Lee[3,4,5], Wen-Kuan Huang[1,6], Chiao-En Wu[1,7], Hsien-Kun Chang[1], Yin-Kai Chao[8], Chen-Kan Tseng[9], Tzu-Keng Chiu[2], Nina Ming-Jung Lin[1], Siou-Ru Ye[1], Jane Ying-Chieh Lee[1], Chia-Hsun Hsieh[1,10*]

[1] Circulating Tumour Cell Lab, Division of Medical Oncology, Department of Internal Medicine, Chang Gung Memorial Hospital at Linkou, College of Medicine, Chang Gung University, Taoyuan, Taiwan.

[2] Graduate Institute of Biochemical and Biomedical Engineering, Chang Gung University, Taoyuan, Taiwan.

[3] Division of Endocrinology and Metabolism, Department of Internal Medicine, Taichung Veterans General Hospital, Taichung, Taiwan

[4] Department of Public Health, College of Public Health, China Medical University, Taichung, Taiwan

[5] Department of Medical Research, Taichung Veterans General Hospital, Taiwan

[6] Department of Oncology–Pathology, Karolinska Institutet, Stockholm, Sweden; Cancer Center Karolinska, Karolinska University Hospital, Stockholm SE-17176, Sweden

[7] Northern Institute for Cancer Research, School of Medicine, Newcastle University, Newcastle, United Kingdom.

[8] Division of Thoracic Surgery, Chang Gung Memorial Hospital, College of Medicine, Chang Gung University, Taoyuan, Taiwan

[9] Department of Radiation Oncology, Chang Gung Memorial Hospital, College of Medicine, Chang Gung University, Taoyuan, Taiwan

[10] Department of Chemical and Materials Engineering, Chang Gung University, Taoyuan, Taiwan.
ǂ Po-Jung Su, Min-Hsien Wu, and Hung-Ming Wang contributed equally to this study.

Experiments for Efficiency of Detection and Patients' Sample Analysis

Four mL peripheral blood drawn from healthy individuals was spiked with and without 1000 OECM1 cells. Peripheral blood mononuclear cells (PBMCs), including epithelial cells and spiked OECM1 cells, were isolated by density gradient centrifugation (30 min, 1500 rpm). Samples were negatively enriched by adding EasySep CD45 Depletion Cocktail (STEMCELL Technologies Inc., Vancouver, BC, Canada) at 25 μL/mL cells and EasySep Magnetic Nanoparticles (STEMCELL) at 50 μL/mL cells. Immunomagnetically enriched samples containing spiked OECM1 cells were collected and labelled with fluorescein isothiocyanate (FITC)-conjugated anti-EpCAM monoantibody (1:800 dilution; eBioscience, Inc., San Diego, CA, USA) and PE anti-human CD235a (Glycophorin A) monoantibody (1:100 dilution, BioLegend, San Diego, CA, USA). The performance recovery, which was defined as the division of the number of OECM1 cells detected by flow cytometry (BD FACSCalibur, BD Biosciences, San Jose, CA, USA) by the number of spiked OCEM1 cells, and the coefficient of variation (CV) value were calculated. CTCs were defined as the cells that were positive for EpCAM and negative for both CD45 and CD235a.

LEGEND

Supplementary Figure S1. Correlations among the number of circulating tounour cells (CTC), locations and chemotherapies and the survival impacts of the CTC plus response score (CTCR). Panel A shows that cancer location is not correlated with overall survival. Interestingly, the chemotherapy regimen (paclitaxel

plus carboplatin or cisplatin plus 5-fluorouracil [5FU]) does not influence overall survival but showed a trend toward extending survival in the paclitaxel plus carboplatin group compared with that of the cisplatin plus 5FU group (Panels B and C). In panel D, the higher the circulating tumour cells plus response (CTCR) score, the shorter the disease-specific progression-free survival seems to be, with a log rank test P value of < 0.001 by Kaplan-Meier survival analysis.

Supplementary Table

Supplementary Table S1. The coordinates of the curve and the area under the curve.

Test Result Variable(s): CTC

Positive if ≥ [a]	Sensitivity	1 - Specificity	Youden test
14.330	.614	.400	1.214
15.015	.614	.350	1.264
16.100	.596	.350	1.246
17.725	.579	.350	1.229
18.875	.579	.300	1.279
19.500	.544	.300	1.244
20.375	.526	.300	1.226
20.875	.526	.250	1.276
**21.000**	**.526**	**.200**	**1.326**
21.400	.509	.200	1.309
21.550	.491	.200	1.291
21.650	.474	.200	1.274
22.000	.456	.200	1.256

22.550	.456	.150	1.306
22.900	.439	.150	1.289
23.400	.404	.150	1.254
24.775	.386	.150	1.236
26.250	.386	.100	1.286
27.025	.386	.000	1.214
27.800	.368	.000	1.264
28.900	.351	.000	1.246

Test Result Variable(s): CTC

Area	Std. Error[a]	Asymptotic Sig.[b]	Asymptotic 95% Confidence Interval	
			Lower Bound	Upper Bound
.655	.062	.040	.535	.776

The test result variable(s): CTC has at least one tie between the positive actual state group and the negative actual state group. Statistics may be biased.

a. Under the nonparametric assumption

b. Null hypothesis: true area = 0.5

Supplementary Table S2. Correlations Among CTC Number Category and First Responses to CCRT and Patterns of Failure.

		CTC category		
		CTC number < 21 cells/mL	CTC number ≥ 21 cells/mL	chi-square P value
Response to CCRT	Complete remission (CR)	3	1	
	Partial response (PR)	19	10	
	Stable disease (SD)	4	1	
	Progressive disease (PD)	0	11	
	Not available (N/A)	2	6	0.001
Pattern of Failure	Locoregional progression	6	13	
	Distant failure/progression	6	9	
	Second primary tumour	3	0	
	No disease-specific progression	13	7	0.027

Abbreviations: CTC, circulating tumour cells; CR, complete remission; PR, partial regression; SD, stable disease; PD, progressive disease; N/A, not available.

Supplementary Figure

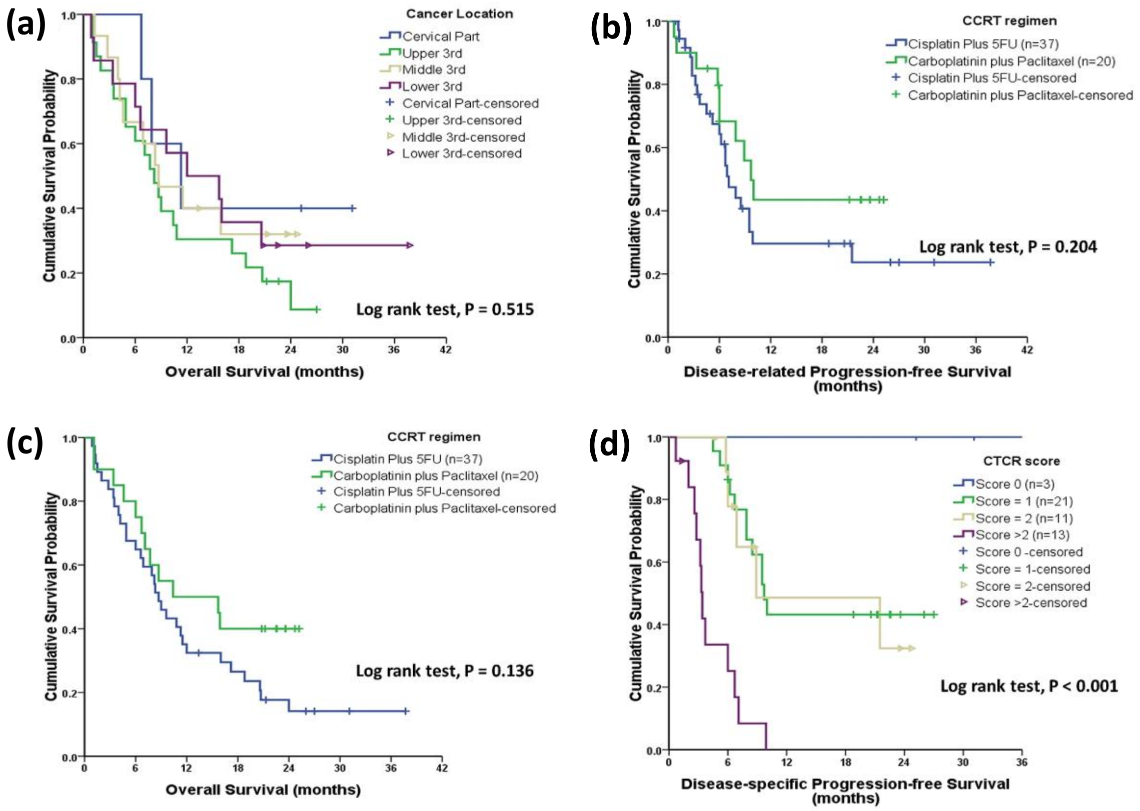

Supplementary Figure S1. Correlations among the number of circulating tumour cells (CTC), locations and chemotherapies and the survival impacts of the CTC plus response score (CTCR).
